# The effect of prior statin use on 30-day mortality for patients hospitalized with community-acquired pneumonia

**DOI:** 10.1186/1465-9921-6-82

**Published:** 2005-07-25

**Authors:** Eric M Mortensen, Marcos I Restrepo, Antonio Anzueto, Jacqueline Pugh

**Affiliations:** 1 VERDICT Research Center, Audie L Murphy VA Hospital, San Antonio, Texas, USA; 2 Division of General Medicine, The University of Texas Health Science Center at San Antonio, USA; 3 Division of Pulmonary and Critical Care Medicine, The University of Texas Health Science Center at San Antonio, USA

## Abstract

**Background:**

Recent studies suggest that HMG-CoA reductase inhibitors ("statins") may have beneficial effects for patients at risk for some types of infections. We examined the effect of prior outpatient use of statins on mortality for patients hospitalized with community-acquired pneumonia.

**Methods:**

A retrospective cohort study conducted at two tertiary teaching hospitals. Eligible subjects were admitted with a diagnosis of, had a chest x-ray consistent with, and had a discharge ICD-9 diagnosis of pneumonia. Subjects were excluded if they were "comfort measures only" or transferred from another acute care hospital. Subjects were considered to be on a medication if they were taking it at the time of presentation.

**Results:**

Data was abstracted on 787 subjects at the two hospitals. Mortality was 9.2% at 30-days and 13.6% at 90-days. At presentation 52% of subjects were low risk, 34% were moderate risk, and 14% were high risk based on the pneumonia severity index. In the multivariable regression analysis, after adjusting for potential confounders including a propensity score, the use of statins at presentation (odds ratio 0.36, 95% confidence interval 0.14–0.92) was associated with decreased 30-day mortality.

**Discussion:**

Prior outpatient statin use was associated with decreased mortality in patients hospitalized with community-acquired pneumonia despite their use being associated with comorbid illnesses likely to contribute to increased mortality. Confirmatory studies are needed, as well as research to determine the mechanism(s) of this protective effect.

## Background

Community-acquired pneumonia is the seventh leading cause of death and the leading cause of infectious death in the United States [1]. Although mortality due to community-acquired pneumonia decreased significantly with the introduction of antibiotics in the 1950s, since that time mortality has been stable or increasing [2]. Despite this, only a few new classes of antibiotics have been added to the armamentarium for treating community-acquired pneumonia in the last 20 years and no new classes of medications beyond antibiotics have been added since the 1950s.

Recent studies have demonstrated that inhibitors of HMG-CoA reductase ("statins") have significant immunomodulatory effects and reduce systemic cytokine levels [3-8]. These cytokines play an important role in host defense mechanisms for patients with community-acquired pneumonia but under certain conditions may lead to septic shock or acute respiratory distress syndrome (ARDS) [9-11]. Recent studies have demonstrated that in patients hospitalized with bacteremia or diabetic lower extremity infections those patients who were taking statins had a significantly decreased odds of death after adjusting for other potential confounders [12,13].

The study aim was to assess the effects of prior outpatient statin use on 30-day mortality for patients hospitalized with community-acquired pneumonia after adjusting for other potential confounders including a propensity score based upon the use/non-use of statins at presentation.

## Methods

This a retrospective cohort study of patients hospitalized with community-acquired pneumonia at 2 academic tertiary care hospitals in San Antonio, Texas. Both hospitals are teaching affiliates of the University of Texas Health Science Center at San Antonio. The Institutional Review Board of the University Health Science Center at San Antonio approved the research protocol with exempt status.

### Study Sites/Inclusion and Exclusion Criteria

We identified all patients admitted to the study hospitals between January 1, 1999 and December 1, 2002 with a primary discharge diagnosis of pneumonia (ICD-9 codes 480.0–483.99 or 485–487.0) or secondary discharge diagnosis of pneumonia with a primary diagnosis of respiratory failure (518.81) or sepsis (038.xx). Subjects were included if they were 1) greater than 18 years of age, 2) had an admission diagnosis of community-acquired pneumonia, and 3) had a radiographically confirmed infiltrate or other finding consistent with community-acquired pneumonia on chest x-ray or CT obtained within 24 hours of admission.

Exclusion criteria included 1) having been discharged from an acute care facility within 14 days of admission, 2) transfer after being admitted to another acute care hospital, and 3) being comfort measures only on this admission. If a subject was admitted more than once during the study period, only the first hospitalization was abstracted.

### Data Abstraction

Chart review data included: demographics, comorbid conditions, physical examination findings, laboratory data, and chest radiograph reports. In addition, data on important processes of care measures for patients hospitalized with community-acquired pneumonia were also abstracted: first dose of antibiotics within 4 hours and 8 hours of admission, collection of blood cultures prior to antibiotic administration, and obtaining blood cultures and oxygen saturation measurement within 24 hours of presentation [14]. Antimicrobial therapy was considered guideline-concordant if it agreed with either the 2000 Infectious Diseases Society of America or 2001 American Thoracic Society guidelines [15,16]. Information on all outpatient medications that were either 1) reported as currently being taken by the patient at presentation, or 2) listed in the electronic medical record, were recorded. Patients were defined as taking a statin if they had a statin listed on the electronic medical record (as an outpatient medication) or history and physical under outpatient medications.

Mortality was assessed using information from the Texas Department of Health and Department of Veteran Affairs clinical database. Mortality status was assessed through December 2002.

### Risk Adjustment

The pneumonia severity index score was used to assess severity of illness at presentation [17]. The pneumonia severity index is a validated prediction rule for 30-day mortality in patients with community-acquired pneumonia. This rule is based on three demographic characteristics, five comorbid illnesses, five physical examination findings, and seven laboratory and radiographic findings from the time of presentation. Patients are classified into five risk classes with 30-day mortality ranging from 0.1% for class I to 27% for class V for patients enrolled in the PORT cohort study [17].

### Outcome

We used 30-day mortality as the outcome for this study. Previous research has demonstrated that 30-day mortality is primarily due to the community-acquired pneumonia rather than other co-existing co-morbid conditions [18,19]. Therefore by using 30-day mortality as our outcome we are able to minimize the effect of statin use on other co-morbid conditions.

### Sample Size

Sample size calculations were based on an assumption of a 30% overall utilization of statins and a 40% difference in use between those who died and survived. We calculated that 800 subjects were needed to have an 80% probability to detect a significant mortality difference at 30-days (with an α of 0.05 and β of 0.20).

### Statistical Analyses

Univariate statistics were used to test the association of sociodemographic and clinical characteristics with all-cause 30-day mortality. Categorical variables were analyzed using the Chi-square test and continuous variables were analyzed using Student's t-test.

A propensity score technique was used to balance covariates associated with statin use between groups [20]. The use of the propensity score technique provides a way, in non-randomized studies, to control for pretreatment differences by defining sets of comparable patients. The propensity score was derived from a logistic regression model. A dichotomous indicator variable indexing whether a patient was on a statin was our response variable. The covariates used in the propensity score model were the pneumonia severity index score (which includes comorbid conditions such as congestive heart failure, liver disease, and history of stroke), history of alcoholism, history of diabetes mellitus, coronary artery disease, and current tobacco use. Variables were entered, and maintained, in the model if they had a p-value <0.20 in the univariate analysis (with statin use as the dependent variable) and had a p-value <0.20 in the final model.

We used a Cox proportional hazard model to estimate, and graph, the baseline survivor functions after adjusting for the propensity score and processes of care including use of guideline-concordant antibiotics, initial dose of antibiotics within 4 hours, obtaining blood cultures prior to antibiotics and within 24 hours, and assessing oxygenation at presentation.

A multivariable logistic regression model was derived with 30-day mortality as the dependent variable, and the propensity score, use of statin at presentation, and process of care measures (initial antibiotics within 4 hours and obtaining blood cultures prior to initial dose of antibiotics, and whether antimicrobial therapy was guideline concordant) as potential confounding variables. Interactions were assessed using cross-product terms between the medications and all of the other variables retained in the models. No significant interactions terms were noted, so they were excluded from the final models. All analyses were performed using STATA version 8 (Stata Corporation, College Station, Texas).

## Results

Data was abstracted on 787 patients at the two hospitals. The mean age was 60 years with a standard deviation of 16 years. The population was 79% male, 84% were admitted through the emergency department, and 20% were admitted to the intensive care unit (ICU) within the first 24 hours after admission. Mortality was 9.2% at 30-days and 13.6% at 90-days. By pneumonia severity index, 52% were low risk (pneumonia severity index classes I-III), 34% were moderate risk (pneumonia severity index class IV), and 14% were high risk (pneumonia severity index class V). Regarding community-acquired pneumonia-related processes of care, 28% received the initial dose of antibiotics within 4 hours of presentation and an additional 22% received the initial antibiotic dose within 8 hours, 76% of patients had blood cultures obtained within 24 hours and prior to antibiotics, and oxygenation was assessed at presentation in 91%.

Table 1 shows the demographic factors, clinical characteristics, and processes of care data for this population by 30-day mortality. In the univariate analysis numerous individual components of the PSI were significantly associated with 30-day mortality including age, nursing home residency, history of congestive heart failure, history of malignancy, altered mental status, systolic blood pressure < 90 mmHg, tachycardia> 125 beats per minute, arterial acidosis, elevated blood urea nitrogen 30 mg/dl, serum sodium < 130 meq/l, and pleural effusion on chest radiograph. The only processes of care that were statistically significant were the assessment of oxygenation within 24 hours or presentation and use of guideline-concordant antibiotics. Statin use had only a borderline significance (p = 0.07) in the univariate analysis.

**Table 1:** Subject Demographic and Clinical Characteristics by 30-Day Mortality*

**Variable**	**30-Day Mortality**		
	**Alive (n= 715)**	**Dead (n= 72)**	**p-valzue**
Age, years +/- standard deviation	60.2+/-16.4	62.9 +/-16.4	0.09
Men	561 (79)	60 (83)	0.3
Nursing home resident	41 (6)	13 (18)	<0.001
Admitted through emergency department	598 (84)	58 (81)	0.5
Admitted to intensive care within 24 hours	118 (17)	36 (50)	<0.001
***Preexisting Comorbid Conditions***			
Congestive heart failure	105 (15)	18 (25)	0.02
Chronic pulmonary disease	195 (27)	23 (31)	0.4
History of stroke	93 (13)	12 (17)	0.4
Chronic liver disease	83 (12)	11 (15)	0.4
History of malignancy	58 (8)	20 (28)	<0.001
Renal insufficiency	74 (10)	13 (18)	0.05
***History, Physical, Laboratory, and Radiographic Data***			
Altered mental status	68(10)	17(24)	<0.001
Respiratory rate > 30 per minute	71 (10)	11 (15)	0.2
Systolic blood pressure < 90 mmHg	16 (2)	5 (7)	0.02
Heart rate > 125 per minute	86 (12)	19 (26)	0.001
Temperature < 95° or > 104°F	19 (3)	2 (3)	0.9
Arterial pH < 7.35	37 (5)	12 (17)	<0.001
Arterial oxygenation saturation < 90%	149 (21)	27 (38)	0.001
Hematocrit < 30%	64 (9)	8 (11)	0.5
Serum blood urea nitrogen > 30 mg/dL	135 (19)	33 (46)	<0.001
Serum glucose > 250 mg/dL	71 (10)	5 (7)	0.4
Serum sodium < 130 meq/L	98 (14)	18 (25)	0.01
Pleural effusion on chest radiograph	160 (11)	29 (35)	0.001
***Pneumonia Severity Index***			
Class I-III	393 (54)	16 (22)	
Class IV	240 (34)	26 (36)	
Class V	82 (12)	30 (42)	<0.001
***Processes of Care***			
Initial antibiotics within 4 hours	201 (28)	22 (31)	0.7
Initial antibiotics within 8 hours	358 (50)	36 (50)	1.0
Blood cultures prior to antibiotics	540 (76)	55 (76)	0.9
Oxygenation assessed ≤ 24 hours	538 (75)	65 (90)	0.004
Guideline-concordant antibiotics used	574 (80)	51 (71)	0.05
***Outpatient Medications***			
Statin	105 (15)	5 (7)	0.07

* Data are presented as number (%) or mean +/-standard deviation

**Figure 1:**
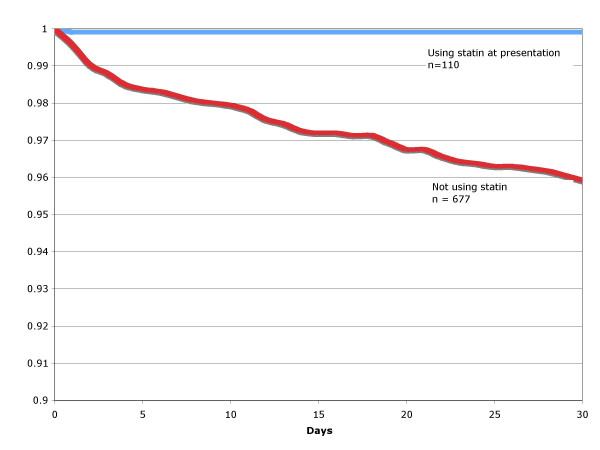
Proportion of surviving patients hospitalized with community-acquired pneumonia by use of statin versus non-use after adjusting for the propensity score and other potential confounders (p = 0.001).

Of the 787 subjects, 110 subjects (14%) were on statins at presentation. Table 2 demonstrates the association between clinical and demographic variables and the use/non-use of statins. Components of the PSI that were significantly associated with statin use include increased age, history of congestive heart failure, history of stroke, systolic blood pressure < 90 mmHG, and an elevated serum glucose. History of diabetes mellitus was also associated with statin use. Conditions inversely associated with statin use include: nursing home residence, history of alcoholism, chronic liver disease, current tobacco use, and pleural effusion on chest x-ray. Figure 1 displays the % survival by statin-use versus non-use over 30-days after adjusting for the propensity score and processes of care, showing that statin use is associated with higher survival at 30-days (p = 0.001).

**Table 2:** Use versus non-use of statin by demographic and clinical characteristics*

**Variable**	**Statin**		
	**Not on statin (n = 677)**	**On statin (n = 110)**	**p-value**
Age, years +/- standard deviation	59.4+/-16.8	66.3+/-12.3	<0.001
Men	529(78)	92(84)	0.2
Nursing home resident	51(8)	3(3)	0.06
Admitted through emergency department	570(84)	86(78)	0.1
Admitted to intensive care within 24 hours	135(20)	19(17)	0.5
***Preexisting Comorbid Conditions***			
Diabetes Mellitus	168(25)	62(56)	<0.001
Alcoholism	79(12)	5(5)	0.03
Current tobacco use	217(32)	18(16)	0.001
Congestive heart failure	99(15)	24(22)	0.05
History of stroke	78(12)	27(25)	<0.001
Chronic liver disease	91(13)	3(3)	0.001
History of malignancy	68(10)	10(9)	0.8
Renal insufficiency	74(11)	13(12)	0.8
***History, Physical, Laboratory, and Radiographic Data***			
Altered mental status	76(11)	9(8)	0.3
Respiratory rate > 30 per minute	69(10)	13(11)	0.6
Systolic blood pressure < 90 mmHg	21(3)	0(0)	0.06
Heart rate > 125 per minute	96(14)	9(8)	0.09
Temperature < 95° or > 104°F	17(3)	4(4)	0.5
Arterial pH < 7.35	39(6)	10(9)	0.2
Arterial oxygenation saturation < 90%	151(22)	25(22)	0.9
Hematocrit < 30%	65(10)	7(6)	0.3
Serum blood urea nitrogen > 30 mg/dL	146(22)	22(20)	0.7
Serum glucose > 250 mg/dL	58(9)	18(16)	0.01
Serum sodium < 130 meq/L	105(15)	11(10)	0.13
Pleural effusion on chest radiograph	172(25)	17(15)	0.02
***Pneumonia Severity Index***			
Class I-III	357(53)	52(47)	
Class IV	222(33)	44(40)	
Class V	98(14)	14(13)	0.3

* Data are presented as number (%) or mean +/-standard deviation

In the multivariable regression analysis, after adjusting for the propensity score and processes of care, the use of statins at presentation (odds ratio 0.36, 95% confidence interval 0.14–0.92) was significantly associated with decreased 30-day mortality.

## Discussion

We found that prior outpatient use of statins was associated with decreased 30-day mortality for subjects hospitalized with community-acquired pneumonia. Our findings provide further support to previous work that demonstrate that statin use is associated with decreased mortality for patients with acute bacterial illnesses [12,13]. Further studies are needed to examine the impact of statins, both pre-hospitalization and acute, on patients hospitalized with community-acquired pneumonia and other bacterial illnesses.

Our study, with a methodologically stronger cohort design, supports the findings of the recent case-control study which demonstrated that patients hospitalized with bacteremia who were on statins at admission had a significant reduction in in-hospital mortality (28% versus 6%, p<0.002) [12]. In the multivariate analysis, after adjustment for confounding factors (including comorbid conditions, age, concurrent medications, site of infection, vital signs, and laboratory data) not being on a statin (odds ratio 7.6, 95% confidence interval 1.01–57.5) was significantly associated with mortality. This prior research, combined with our results, supports the need for further research to examine the impact of statins in the treatment of infectious diseases.

Although our study was retrospective and subject to the recognized limitations of such studies, we carefully assembled our cohort from complete patient discharge data to avoid ascertainment bias. Additionally, during chart abstraction we encountered a very small amount (<5%) of missing data. Our sample was predominantly men due to the inclusion of a VA hospital and it is possible, but unlikely, that women may have differential responsiveness to statins as compared to men. Also we are unable to assess factors such as duration of statin use, inpatient continuation of the statin, or the dose effect due to the design of this study. In addition we are unable to control for quality of health care that patients had prior to hospitalization. Further research is needed to examine these factors. Finally, as in any non-experimental study, we are unable to state conclusively that the prior outpatient use of statin is the cause of decreased mortality in this cohort. However, since patients on statins have numerous medical conditions that are significantly associated with increased short-term morality we feel that we have good evidence that these medications may have beneficial effects for patients hospitalized with community-acquired pneumonia.

## Conclusion

Our study finds that prior outpatient use of statins reduces mortality for patients hospitalized with community-acquired pneumonia. Our results add an additional potential benefit of statin use to the already compelling data for their use in patients with coronary artery disease, hypercholesterolemia, diabetes, and peripheral vascular disease. Additionally, patients with diabetes and vascular disease are at higher risk for either contracting pneumonia or dying from pneumonia when they do contract it. Further studies are needed to confirm the magnitude of the impact of statins, either pre-hospitalization or acute, on patients hospitalized with community-acquired pneumonia and to elucidate the mechanism by which they may work.

## Competing interests

None of the authors, except for Dr. Anzueto, have any conflicts of interests to disclose regarding this paper. Dr. Anzueto is currently a consultant with Pfizer, Ortho-McNeil, and Bayer Pharma.

## Authors' contributions

EMM originated and coordinated the study, obtained funding, contributed to the analysis of the data, and preparation of the paper.

MIR contributed to the design of the study, contributed to the analysis of the data, and preparation of the paper.

AA contributed to the design of the study and preparation of the paper.

JP contributed to the design of the study, contributed to the analysis of the data, and preparation of the paper.

## References

[B1] Hoyert DL, Arias E, Smith BL ( 2001). Deaths: Final Data for 1999. Natl Vital Statistics Report.

[B2] Gilbert K, Fine MJ (1994). Assessing prognosis and predicting patient outcomes in community-acquired pneumonia. Seminars in Respiratory Infections.

[B3] Jialal I, Stein D, Balis D, Grundy SM, Adams-Huet B, Devaraj S (2001). Effect of hydroxymethyl glutaryl coenzyme a reductase inhibitor therapy on high sensitive C-reactive protein levels. Circulation.

[B4] Musial J, Undas A, Gajewski P, Jankowski M, Sydor W, Szczeklik A (2001). Anti-inflammatory effects of simvastatin in subjects with hypercholesterolemia. International Journal of Cardiology.

[B5] de Bont N, Netea MG, Rovers C, Smilde T, Demacker PN, van der Meer JW, Stalenhoef AF (1998). LPS-induced cytokine production and expression of LPS-receptors by peripheral blood mononuclear cells of patients with familial hypercholesterolemia and the effect of HMG-CoA reductase inhibitors. Atherosclerosis.

[B6] Rosenson RS, Tangney CC, Casey LC (1999). Inhibition of proinflammatory cytokine production by pravastatin. Lancet.

[B7] Ridker PM, Rifai N, Pfeffer MA, Sacks FM, Moye LA, Goldman S, Flaker GC, Braunwald E (1998). Inflammation, pravastatin, and the risk of coronary events after myocardial infarction in patients with average cholesterol levels. Cholesterol and Recurrent Events (CARE) Investigators. Circulation.

[B8] Strandberg TE, Vanhanen H, Tikkanen MJ (1999). Effect of statins on C-reactive protein in patients with coronary artery disease. Lancet.

[B9] Moussa K, Michie HJ, Cree IA, McCafferty AC, Winter JH, Dhillon DP, Stephens S, Brown RA (1994). Phagocyte function and cytokine production in community acquired pneumonia.. Thorax.

[B10] Puren AJ, Feldman C, Savage N, Becker PJ, Smith C (1995). Patterns of cytokine expression in community-acquired pneumonia. Chest.

[B11] Bauer TT, Monton C, Torres A, Cabello H, Fillela X, Maldonado A, Nicolas JM, Zavala E (2000). Comparison of systemic cytokine levels in patients with acute respiratory distress syndrome, severe pneumonia, and controls. Thorax.

[B12] Liappis AP, Kan VL, Rochester CG, Simon GL (2001). The effect of statins on mortality in patients with bacteremia. Clinical Infectious Diseases.

[B13] Seraphin LM, Liappis AP, Kan VL, Simon GL (2001). Increased incidence of lower extremity infections among diabetic patients receiving statins.: September and Decmeber 2001..

[B14] Meehan TP, Fine MJ, Krumholz HM, Scinto JD, Galusha DH, Mockalis JT, Weber GF, Petrillo MK, Houck PM, Fine JM (1997). Quality of care, process, and outcomes in elderly patients with pneumonia. JAMA.

[B15] Niederman MS, Mandell LA, Anzueto A, Bass JB, Broughton WA, Campbell GD, Dean N, File T, Fine MJ, Gross PA, Martinez F, Marrie TJ, Plouffe JF, Ramirez J, Sarosi GA, Torres A, Wilson R, Yu VL (2001). Guidelines for the management of adults with community-acquired pneumonia. Diagnosis, assessment of severity, antimicrobial therapy, and prevention. Am J Respir Crit Care Med.

[B16] Bartlett JG, Dowell SF, Mandell LA, File Jr TM, Musher DM, Fine MJ (2000). Practice guidelines for the management of community-acquired pneumonia in adults. Infectious Diseases Society of America. Clin Infect Dis.

[B17] Fine MJ, Auble TE, Yealy DM, Hanusa BH, Weissfeld LA, Singer DE, Coley CM, Marrie TJ, Kapoor WN (1997). A prediction rule to identify low-risk patients with community-acquired pneumonia. N Engl J Med.

[B18] Mortensen EM, Kapoor WN, Chang CC, Fine MJ (2003). Assessment of mortality after long-term follow-up of patients with community-acquired pneumonia. Clin Infect Dis.

[B19] Mortensen EM, Coley CM, Singer DE, Marrie TJ, Obrosky DS, Kapoor WN, Fine MJ (2002). Causes of death for patients with community-acquired pneumonia: results from the Pneumonia Patient Outcomes Research Team cohort study. Arch Intern Med.

[B20] Stone RA, Obrosky DS, Singer DE, Kapoor WN, Fine MJ (1995). Propensity score adjustment for pretreatment differences between hospitalized and ambulatory patients with community-acquired pneumonia. Pneumonia Patient Outcomes Research Team (PORT) Investigators. Med Care.

